# Fully Integrated Ultrathin Solid Immersion Grating Microspectrometer for Handheld Visible and Near‐Infrared Spectroscopic Applications

**DOI:** 10.1002/advs.202304320

**Published:** 2023-10-17

**Authors:** Jung‐Woo Park, Jaehun Jeon, Gi Beom Kim, Ki‐Hun Jeong

**Affiliations:** ^1^ Department of Bio and Brain Engineering Korea Advanced Institute of Science and Technology (KAIST) 291 Daehak‐ro Yuseong‐gu Daejeon 34141 Republic of Korea; ^2^ KAIST Institute for Health Science and Technology (KIHST) Korea Advanced Institute of Science and Technology (KAIST) 291 Daehak‐ro Yuseong‐gu Daejeon 34141 Republic of Korea

**Keywords:** microspectrometer, ripeness prediction, solid immersion grating, spectral analysis

## Abstract

Despite advances in microfabrication, compact spectrometers still face challenges in shrinking their size without sacrificing optical performance. Here,  the solid immersion grating microspectrometer (SIG‐µSPEC) for high spectral resolution in a broad operational wavelength range is reported. The spectroscopic module incorporates a silicon microslit, index‐matched lens, plane mirrors, solid immersion grating (SIG), and a CMOS line sensor within a small form factor. The SIG facilitates high angular dispersion of light on a planar focal plane, resulting in an average spectral resolution of 5.8 nm, with over 76% maximum sensitivity from 400 to 800 nm. SIG‐µSPEC measures the spectral reflectance of fruits at different ripening stages, clearly revealing changes in the chlorophyll absorption band. The measured spectrum is further utilized for the precise prediction of the soluble solid content (SSC) levels, achieving a high correlation (R^2^ = 0.91) and a ratio of prediction‐to‐deviation of 2.36. This compact microspectrometer holds the potential for precise and non‐invasive spectral analysis across point‐of‐care fields.

## Graphical abstract

This study reports a compact and high‐resolution solid immersion grating (SIG) microspectrometer. SIG, along with an index‐matched convex lens, allows high angular dispersion. SIG‐µSPEC provides a high spectral resolution of 5.8 nm over a broad operational range. SIG‐µSPEC also predicts precise solid soluble content levels, with a high R2 of 0.91 and a ratio of prediction‐to‐deviation of 2.36. This compact microspectrometer holds the potential for precise spectral analysis across point‐of‐care fields.

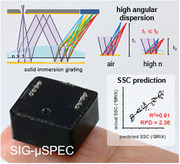

## 1 Introduction

Spectral information is crucial for precise evaluation and identification of materials and substances including chemical analysis, molecular detection, and biomedical diagnosis.^[^ ^1^, ^2^, ^3^ ^]^ Spectrometers achieve ultra‐high resolution over a broad spectral range through the precise arrangement of dispersive optical components.^[^ ^4^ ^]^ They consequently provide non‐invasive monitoring in assorted industrial and biomedical applications such as quality control,^[^ ^5^, ^6^, ^7^ ^]^ drug screening,^[^ ^8^ ^]^ and clinical diagnosis.^[^ ^9^, ^10^ ^]^ Despite the broad availability, conventional spectrometers still have bulky and complicated configurations, restraining the practical use in point‐of‐care fields.^[^ ^11^, ^12^ ^]^ Recent advances in microfabrication technologies led to the substantial miniaturization of primary optical components, resulting in advanced portables for real‐time monitoring.^[^ ^13^ ^]^ Handheld spectrometers rapidly expand on‐demand applications such as food quality evaluation,^[^ ^14^, ^15^ ^]^ environmental monitoring,^[^ ^16^, ^17^ ^]^ drug anti‐counterfeiting,^[^ ^18^, ^19^ ^]^ and at‐home healthcare assistance.^[^ ^20^, ^21^, ^22^ ^]^

Integrated dispersion components allow compact spectrometers with stable spectra and simplified signal processing, suitable for handheld applications.^[^ ^23^ ^]^ Unlike temporal dispersion, light dispersion through spatial separation directly provides spectral information, particularly beneficial for microspectrometers.^[^ ^24^ ^]^ Spatial dispersion often utilizes waveguide or grating in a confined space.^[^ ^23^, ^25^ ^]^ Waveguide dispersion microspectrometers have recently embraced holographic element,^[^ ^26^, ^27^ ^]^ planar echelle grating,^[^ ^28^ ^]^ or metasurface^[^ ^29^, ^30^ ^]^ as a substitute for diffractive optical elements to confine light through mode coupling. However, the narrow spacing of the waveguide results in an extremely narrow operational range.^[^ ^31^ ^]^ In addition, high‐cost nanofabrication also restricts an effective area for waveguide dispersion, resulting in a significant decrease in optical throughput. In contrast, grating dispersion microspectrometers effectively simplify the optical configuration with concave grating^[^ ^32^, ^33^ ^]^ or grating‐Fresnel lens^[^ ^34^ ^]^ based on out‐of‐plane dispersion. However, angular dispersion from the grating produces a nonplanar focal plane over a line camera in a short optical path, causing a notable reduction in spectral resolution and sensitivity.^[^ ^35^ ^]^ Such configurations face a trade‐off between the module size and the spectral resolution. Microspectrometers still require both high angular dispersion and a planar focal plane for high‐spectral resolution in a broad operational range.

## 2 Results and Discussion

### 2.1 Solid Immersion Grating Microspectrometer

**Figure 1:**
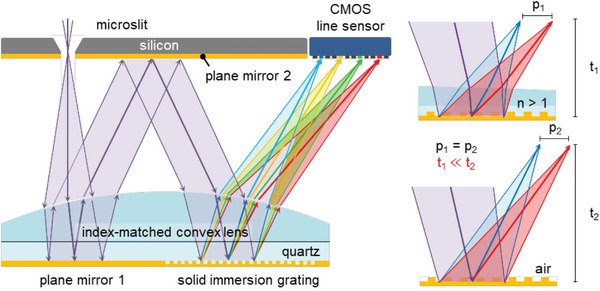
Schematic illustration for ultrathin SIG‐µSPEC. Light enters through a silicon microslit, passing through an index‐matched convex lens, paired plane mirrors, and SIG. The dispersed light is then focused on a CMOS line sensor. SIG along with the convex lens provides high angular dispersion and multispectral planar focal plane on the CMOS line sensor, substantially reducing the thickness of the microspectrometer. The unique configuration results in high resolution and uniform sensitivity for spectrum measurement.

Here, we report the ultrathin solid immersion grating‐based microspectrometer (SIG‐µSPEC) for high spectral resolution and a broad operational range (**Figure** **1**). Light enters the microspectrometer through a silicon microslit, and then passes through an index‐matched convex lens, paired plane mirrors, and solid immersion grating (SIG). The dispersed light is subsequently focused on a CMOS line sensor. The SIG, along with the index‐matched convex lens, allows high angular dispersion on a planar focal plane of the CMOS line sensor, effectively reducing the overall module thickness of the microspectrometer (Figure S1, Supporting Information). This unique configuration yields a high resolution and uniform sensitivity for precise spectrum measurement.

The optical layout of SIG‐µSPEC was designed by using ray‐tracing software (Zemax OpticStudio 16.5, Ansys), considering the physical dimensions of the numerical aperture (0.11 NA), grating period (1.25 µm), and pixel pitch (5.5 µm) of the CMOS line sensor. The operational wavelength ranges from 400 to 800 nm, where the center wavelength at 600 nm surpasses 75% of the maximum intensity (Figure S2, Supporting Information). The SIG features a binary‐phase configuration of 1.25 µm in grating period, 100 nm in height, and 0.4 in duty cycle, based on the numerical calculation (Lumerical FDTD, Ansys) (Figure S3, Supporting Information). Note that the center wavelength at 600 nm exhibits the maximum diffraction efficiency due to the destructive interference at the zeroth order diffraction. The duty cycle was set by considering the critical dimension of the wafer stepper lithography.

**Figure 2:**
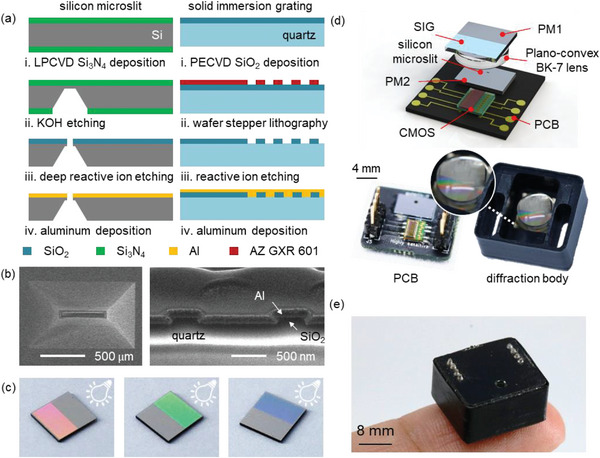
Fully‐packaged SIG‐µSPEC. a) The microfabrication procedure of microslit and SIG. Silicon microslit is fabricated by using wet etching, low‐pressure chemical vapor deposition, deep reactive ion etching, and aluminum deposition. The SIG is separately fabricated by using wafer stepper lithography, plasma enhanced chemical vapor deposition, reactive ion etching, and aluminum deposition. b) A top‐view SEM image of a microslit and a focused ion beam SEM image of the SIG. The microslit has a rectangular aperture of 25 µm × 300 µm, and the SIG has a binary‐phase configuration (period: 1.25 µm, duty cycle: 0.4, height: 100 nm). c) Reflection of the SIG at different illumination angles. d) Micro‐assembled module package of the SIG‐µSPEC, consisting of two primary components: the PCB of a CMOS line sensor, silicon microslit, and PM2, and the diffraction body containing the SIG, PM1, and plano‐convex BK‐7 lens. (e) An optical image of fully‐packaged SIG‐µSPEC with a physical dimension of 8 mm × 12.5 mm × 15 mm.

The silicon microslit and the SIG for the SIG‐µSPEC were microfabricated on a wafer scale using silicon anisotropic wet etching and wafer stepper lithography (**Figure** **2**a). For the silicon microslit, a 150 nm‐thick etch mask of silicon nitride (Si_3_N_4_) layer was deposited on a silicon wafer using low‐pressure chemical vapor deposition (LPCVD) and defined by using dry etching (step i). The silicon wafer was anisotropically etched in a potassium hydroxide (KOH) solution and the silicon nitride layer was completely removed in hydrofluoric acid (step ii). A 1.5 µm‐thick etch mask of silicon dioxide (SiO_2_) was then deposited on the top side of a silicon wafer using plasma enhanced chemical vapor deposition (PECVD). Both the SiO_2_ layer and the silicon were subsequently etched with reactive ion etching (RIE) and deep reactive ion etching (step iii). A 100 nm‐thick aluminum layer was finally deposited on the top silicon surface, serving as a plane mirror 2 (PM2). (step iv). For the SIG, a 100 nm‐thick SiO_2_ layer was deposited on a quartz wafer using PECVD (step i). A positive photoresist (AZ GXR‐601) was spin‐coated on the SiO_2_ layer, and photolithographically defined with wafer stepper lithography (step ii). The SiO_2_ layer was then etched with RIE (step iii) to form diffractive grating structures, and a 100 nm thick aluminum layer was finally deposited, serving as a plane mirror 1 (PM1) and the SIG (step iv). A scanning electron microscopy (SEM) image shows the silicon microslit with a rectangular aperture of 25 µm × 300 µm and an aperture thickness of 30 µm (Figure 2b, left). A focused ion beam SEM image of the SIG shows a binary‐phase configuration of a rectangular SiO_2_ grating with a period of 1.25 µm, duty cycle of 0.4, and height of 100 nm (Figure 2b, right). The SIG exhibits distinct angular dispersion of visible light at different illumination angles (Figure 2c). The center wavelength of the first‐order diffraction is clearly shifted with the illumination angle, resulting in a change in color. The microfabricated silicon microslit and SIG were precisely assembled for the module package of SIG‐µSPEC (Figure 2d). The silicon microslit with PM2 and CMOS line sensor (S13131‐512, Hamamatsu Photonic) were mounted on the printed circuit board (PCB). A plano‐convex BK‐7 lens (LA1470, Thorlab) was permanently glued on the bottom surface of the SIG with UV epoxy resin. Both individual parts were optically aligned and packaged with a matt black housing case using rapid prototyping. A fully‐packaged SIG‐µSPEC finally shows a compact physical dimension of 8 mm × 12.5 mm × 15 mm, comparable to a fingertip size (Figure 2e).

**Figure 3:**
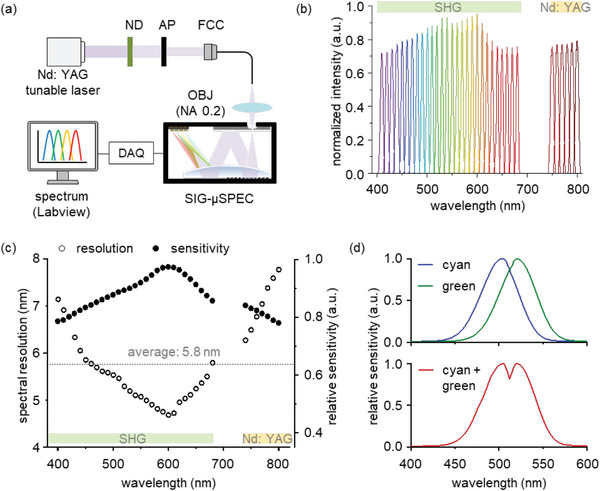
Spectral resolution and relative sensitivity of SIG‐µSPEC. a) Experimental setup for the spectrum measurement involving Nd: YAG tunable laser source, ND, AP, FCC, and OBJ. Hyperspectral laser light (410 – 680 nm, 740 – 800 nm with 10 nm interval) is collected by SIG‐µSPEC. The measured spectrum is then visualized by using Labview DAQmx module. b) Output spectra of tunable laser measured by the SIG‐µSPEC. Each spectrum exhibits a sharp Lorentzian distribution in the spectral range of SHG (410 – 680 nm) and Nd: YAG laser radiation (740 – 800 nm) lines. c) Measured spectral resolution and relative sensitivity. SIG‐µSPEC maintains a sensitivity of over 76% of the maximum while displaying an average spectral resolution of 5.8 nm, with a minimum resolution of 4.7 nm at 600 nm in wavelength. d) Measured spectra of cyan LED, green LED (top, center wavelength, 500 nm, 525 nm each), and simultaneous illumination of two LEDs (bottom). The spectral overlap exhibits two well‐defined peaks that cover 81% of the maximum.

The spectral resolution and the relative sensitivity of the SIG‐µSPEC were evaluated using a Nd: YAG tunable laser (NT341A, EKSPLA) (**Figure** **3**a). Hyperspectral light at 10 nm intervals was injected into the SIG‐µSPEC through an ND filter, aperture (AP), fiber‐coupled collimator (FCC), and objective lens (OBJ) with NA of 0.2. The spectral signal was digitized with a data acquisition (DAQ) board (USB‐6363, National Instrument) at a data rate of 500 kHz over a measurement time of 10 s. The measured spectrum was visualized by using the DAQ software (Labview DAQmx module, National Instrument). The output spectra of the tunable laser were measured with the SIG‐µSPEC and then normalized to the incident light intensity (Figure 3b). Each spectrum clearly exhibits a sharp Lorentzian distribution in the operational range of second harmonic generation (SHG, 410–680 nm) and Nd: YAG laser radiation (740–800 nm) lines. The spectral resolution and relative sensitivity were calculated by using the full‐width half‐maximum (FWHM) and intensity of the spectral peaks (Figure 3c). The SIG‐µSPEC shows an average spectral resolution of 5.8 nm and a minimum of 4.8 nm at 600 nm in wavelength, with a relative sensitivity over 76% of the maximum in the operational range. The experimental results are well‐matched with the calculated values with an average spectral resolution of 5.7 nm and a relative sensitivity over 75% of the maximum (Figure S2b, Supporting Information). The SIG‐µSPEC also effectively suppresses noise from unintended reflections inside the package module, with a low spectral stray light level of −22.8 dB (Figure S4, Supporting Information). Two different LED light of neighboring center wavelengths (HLMP‐CE34‐Y1CDD (cyan: 500 nm), HLMP‐CM3G‐Y10DD (green: 525 nm), Broadco) with FWHMs of 20 nm were simultaneously collected by the SIG‐µSPEC (Figure 3d). Two spectral peaks are well‐defined with an 81% overlap intensity of each maximum, indicating that SIG‐µSPEC allows highly sensitive and accurate measurement of spectra.

**Figure 4:**
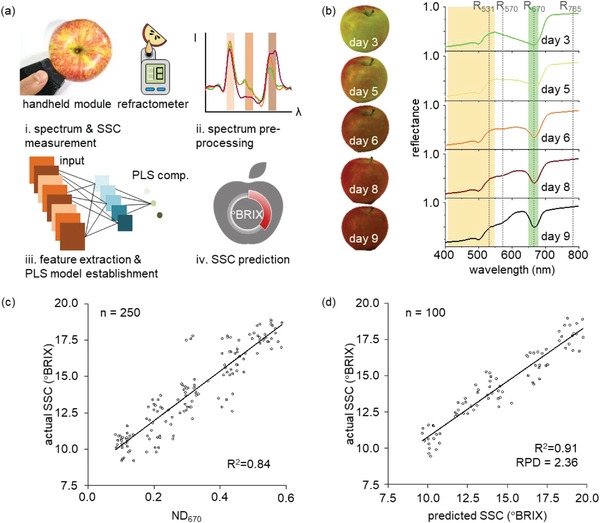
Non‐invasive and quantitative prediction of fruit ripeness using SIG‐µSPEC. a) SSC prediction procedures. SIG‐µSPEC and refractometer measure the reflectance spectrum and SSC level, respectively, which constitute the raw data of apples. A PLSR model is established by using the spectrum pre‐processing and the PLSR model predicts the SSC level after the feature extraction on the measured reflectance spectra. b) Reflectance spectra of apples with different ripening stages. The degradation of chlorophyll increases reflectance at 670 nm, while the spectral absorption of carotenoids remains constant during the apple's ripening. Yellow and green regions indicate the absorption bands of carotenoid and chlorophyll. R_531_, R_570_, R_670_, R_785_ indicate the reflectance at 531, 570, 670, and 785 nm, respectively. c) A linear correlation between the actual SSC level and ND_670_ using the feature selection method. The high‐spectral resolution of SIG‐µSPEC results in a high R‐value (R^2^ = 0.84) (sample size: 250). d) The enhancement of the correlation between predicted and actual SSC levels using the PLSR. The feature extraction method substantially increases R‐value (R^2^ = 0.91) compared to the feature selection method. The RPD also shows 2.36, signifying high accuracy in SSC prediction (sample size: 100).

The SIG‐µSPEC was further utilized for non‐invasive and quantitative spectral analysis to predict fruit ripeness (**Figure** **4**a). The soluble solid content (SSC) level of the apples, i.e., a reliable indicator for fruit ripeness^[^ ^36^ ^]^ was measured from apple extract using a portable digital refractometer (HI‐96811, Hanna Instruments). The reflectance spectra were first measured using the handheld module with SIG‐µSPEC and pre‐processed with the orthogonal signal correction to extract highly correlated features (Figure S5 and Table S1, Supporting Information). The partial least square regression (PLSR) model was then established to predict the SSC level of the apples, which were compared with the actual SSC level. The reflectance spectra of the apple clearly show different spectral intensities depending on the ripening stage (Figure 4b). Chlorophyll exhibits a narrow absorption band (650–690 nm), while carotenoid exhibits a relatively broader absorption band (400–550 nm).^[^ ^37^ ^]^ An increase in reflectance is noticeable between 550 to 690 nm, whereas reflectance remains constant below 550 nm and above 700 nm. Contents of surface pigments are quantitatively analyzed through spectral reflectance using the Sims and Gamon method (Figure S6, Supporting Information).^[^ ^38^ ^]^ Chlorophyll content, indicated as the normalized difference vegetation index at 670 nm (ND_670_), was calculated by ND_670_ = (R_785 –_ R_670_) / (R_785_ + R_670_), where R_785_ and R_670_ are the reflectance at 785 and 670 nm, respectively. ND_670_ significantly decreases from 0.496 to of 0.186 as the apple ripens. The photochemical reflectance index (PRI), indicating the ratio of carotenoid to chlorophyll content, was calculated by PRI = (R_531_ – R_570_) / (R_531_ + R_570_), where R_531_ and R_570_ are the reflectance at 531 and 570 nm, respectively. PRI also decreases from 0.039 to −0.103 as the apple ripens. Note that the degradation of chlorophyll results in a reduction in both indices, affected by the absorption band of chlorophyll.^[^ ^39^ ^]^ The SSC level of the apple was further evaluated through the feature selection method and the feature extraction method, respectively. The feature selection method was further employed for the ND_670_ along with the actual SSC level. The predicted result clearly demonstrates a strong linear correlation with a high R^2^ value of 0.84, indicating that the reflectance of chlorophyll increases as the apples ripen (Figure 4c).^[^ ^40^ ^]^ Note that a high spectral resolution leads to a low standard deviation of the reflectance spectrum, resulting in a high correlation index of R^2^ value.^[^ ^41^ ^]^ The SIG‐µSPEC clearly exhibits high correlation indices compared to the conventional handheld spectrometer (Figure S7, Supporting Information). In addition, the feature extraction method substantially improves the linear correlation by assigning weights and scoring to all the features (Figure 4d). The PLSR model predicts the SSC level of apples based on the extracted features from pre‐processed spectrum data, and then compared with the actual SSC level. A linear correlation between the predicted and the actual SSC levels is further improved up to R^2^ value of 0.91. The ratio of prediction to deviation (RPD) for apple ripeness also shows 2.36, which exceeds the high level‐performance (RPD > 2.0) of the prediction model for spectral analysis.^[^ ^42^ ^]^ Such correlation indices as R^2^, RMSE, and RPD, point toward a higher degree of precision in the prediction of the SSC level, compared to the conventional handheld spectrometer (Figure S8 and Table S2, Supporting Information).

## 3 Conclusion

To conclude, we have successfully demonstrated the SIG‐µSPEC with high‐resolution and high‐sensitivity performance for non‐invasive and quantitative fruit ripeness testing. The SIG‐µSPEC shows high spectral resolution in a broad operational range due to the high angular dispersion, resulting from high refractive index of the SIG. The microspectrometer has a compact physical dimension of 8 mm × 12.5 mm × 15 mm, and an average spectral resolution of 5.8 nm, while maintaining a relative sensitivity over 76% of the maximum. A simple reflectance measurement using SIG‐µSPEC results in precise prediction of SSC level with a high R^2^ value of 0.91 and RPD of 2.36 after feature extraction. Hence, this ultrathin and high‐resolution microspectrometer can serve as a vital tool for precise and non‐invasive measurements in on‐demand agriculture, biomedical, and healthcare applications.

## 4 Experimental Section

### Reflectance Spectrum Measurement of Apples

Reflectance spectra of apples (*Malus Domestica*) were measured with SIG‐µSPEC and white LED (LUW50343‐C1(B), HanyangSemi^™^) at different stages of maturity, ranging from day 1 to day 10. For each apple, five different surface positions were measured with five repetitions for each 10 s. The measured spectrum exhibited an average standard deviation of 2.54% from the reflectance value. The measured spectrum was subtracted from the reference spectrum under a darkroom condition, and the average spectra were visualized by using the Labview DAQmx module with a data rate of 500 kHz.

### Pre‐Processing Tools

Measured reflectance spectra were applied with different pre‐processing tools in order to effectively extract the features. The pre‐processing tools include standard normal variate (SNV), multiplicative scattering correction (MSC), orthogonal signal correction (OSC), and a combination of each method with first derivative. The first derivative was used to suppress the DC noise of the baseline, SNV and MSC were employed to reduce stray light generated from the spectrum measurement while OSC was utilized to remove principal components unrelated to the predicted variable without directly modifying the measured spectrum. OSC was finally applied to the PLSR model with the highest correlation index. All the pre‐processing tools were implemented by using Matlab R2020a (Mathworks^™^).

### PLSR Model Establishment

A PLSR model was established by using extracted features from pre‐processed spectral data in Matlab with PLS Toolbox (Eigenvector Research Inc.). The correlation indices, including R‐square (R^2^), root mean square error (RMSE), and ratio of prediction‐to‐deviation (RPD), were calculated by using the following Equations (1) and (2);

(1) $$\mathrm{RMSE}=\sqrt{\frac{\sum_{i=1}^{m}{\left(y_{i}-{\hat{y}}_{i}\right)}^{2}}{m-1}}$$

where m is the number of test samples, y_*i*_ is the actual SSC level, and $\hat{y_{i}}$ is the predicted SSC level of the apple.

(2) $$\mathrm{RPD}=\frac{\mathrm{SD}}{\mathrm{RMSE}}$$

where SD is the standard deviation of the actual SSC level.

## Conflict of Interest

The authors declare no conflict of interest.

## Supporting information

Supporting Information

## Data Availability

The data that support the findings of this study are available from the corresponding author upon reasonable request.
