# Supplementary material for: Fully Integrated Ultrathin Solid Immersion Grating Microspectrometer for Handheld Visible and Near‐Infrared Spectroscopic Applications
Source: Adv Sci (Weinh). 2023 Oct 17;10(34):2304320. doi: 10.1002/advs.202304320 (PMC10700170; doi:10.1002/advs.202304320)
Supplement: Supplementary file 1 — Supporting Information [file ADVS-10-2304320-s001.pdf]

ADVANCED SCIENCE
Open Access

Supporting Information

for *Adv. Sci.*, DOI 10.1002/advs.202304320

Fully Integrated Ultrathin Solid Immersion Grating Microspectrometer for Handheld Visible and Near-Infrared Spectroscopic Applications

*Jung-Woo Park, Jaehun Jeon, Gi Beom Kim and Ki-Hun Jeong**

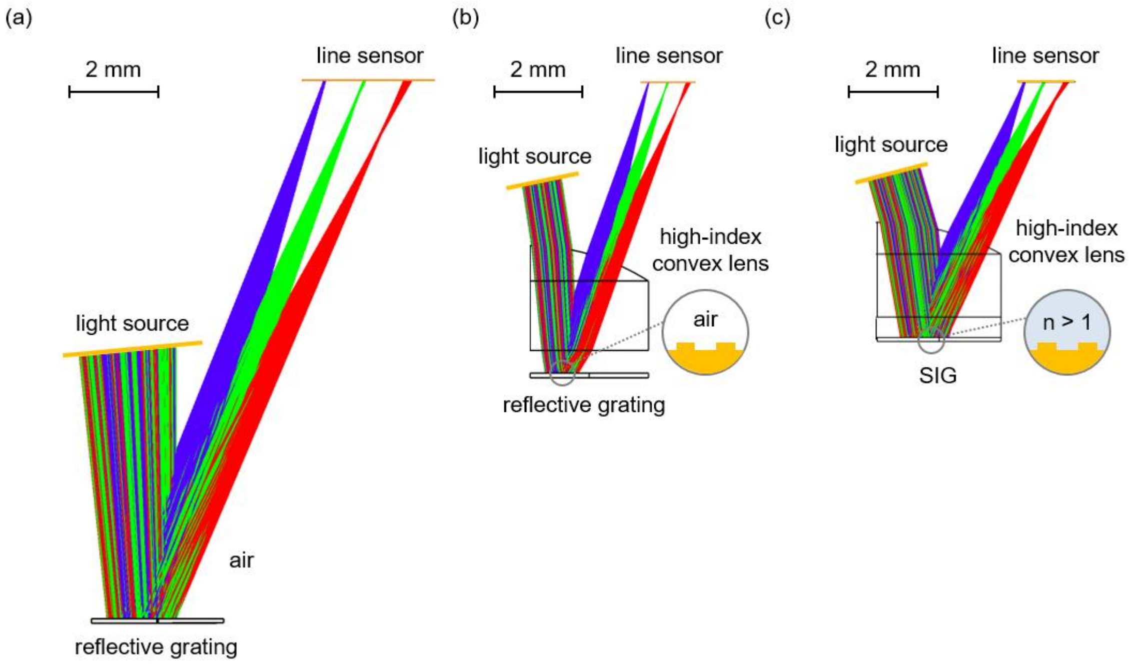

Fig. S1 Optical configuration after light dispersion through different grating structures. Angular dispersion of (a) a reflective grating without a convex lens, (b) a reflective grating with a convex lens, and (c) a SIG with a convex lens using a ray-tracing software on Zemax OpticStudio. Light before SIG are illuminated under same conditions with a cone angle of 2.9 degrees. The height of the spectrometer is 12.2 mm, 7 mm, and 6 mm, respectively. SIG induces high angular dispersion, thereby reduces the optical path length with a high-index convex lens.

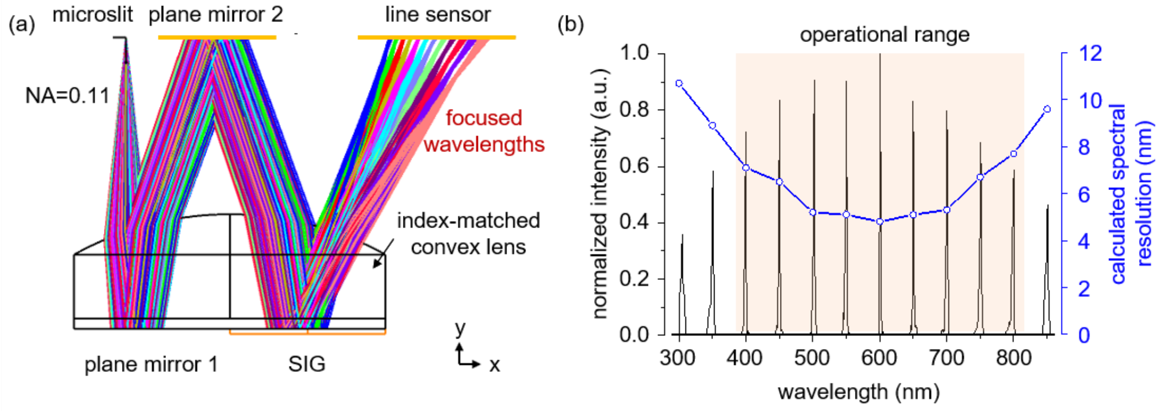

Fig. S2 Optical design of SIG-μSPEC. (a) Optical layout of the SIG-μSPEC using a ray-tracing software on Zemax OpticStudio, where light from 300 to 850 nm with 50 nm interval is focused on the line sensor with a grating period of 1.25 μm. (b) Focal profiles on the image plane and FWHM of the spectral peaks on the line sensor. The operational range of 400 nm to 800 nm is determined by the spectral intensity above 60% of the maximum.

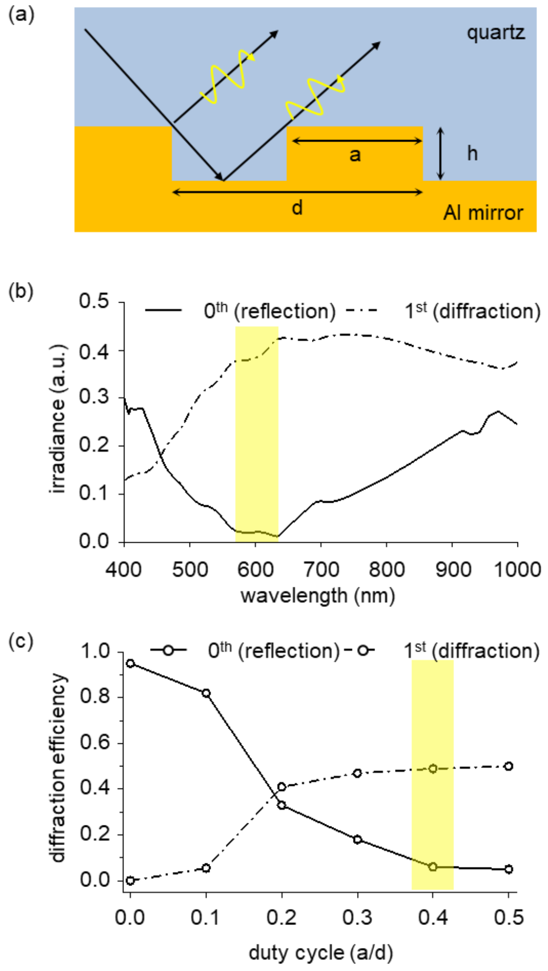

Fig. S3 Optical design of SIG. (a) Schematic illustration of SIG. A grating period of 1.25 μm and a grating height of 100 nm are set to induce a destructive interference of the 0th order diffraction light from the upper and lower planes. (b) Reflectance of the 0th and 1st order diffracted light. The center wavelength is 600 nm, showing the maximum 1st order and the minimum 0th order diffraction. (c) Diffraction efficiency of the 0th and 1st order diffracted light depending on the duty cycle. The duty cycle is optimized at 0.4 by considering the critical dimension of the wafer stepper lithography.

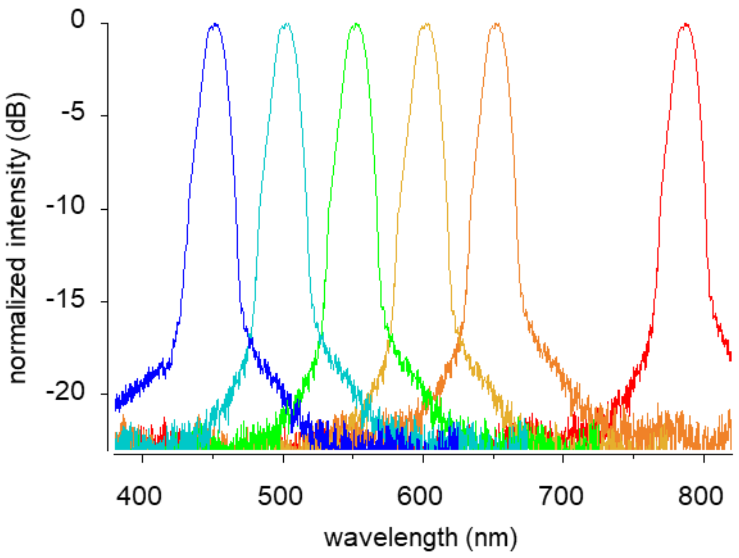

Fig. S4 Spectral stray light level of a fully-packaged SIG- μSPEC depending on the wavelength. The spectral stray light level is determined by the intensity at peak wavelength ±40 nm. The average spectral stray light level ranging from 400 to 800 nm is -22.8 dB.

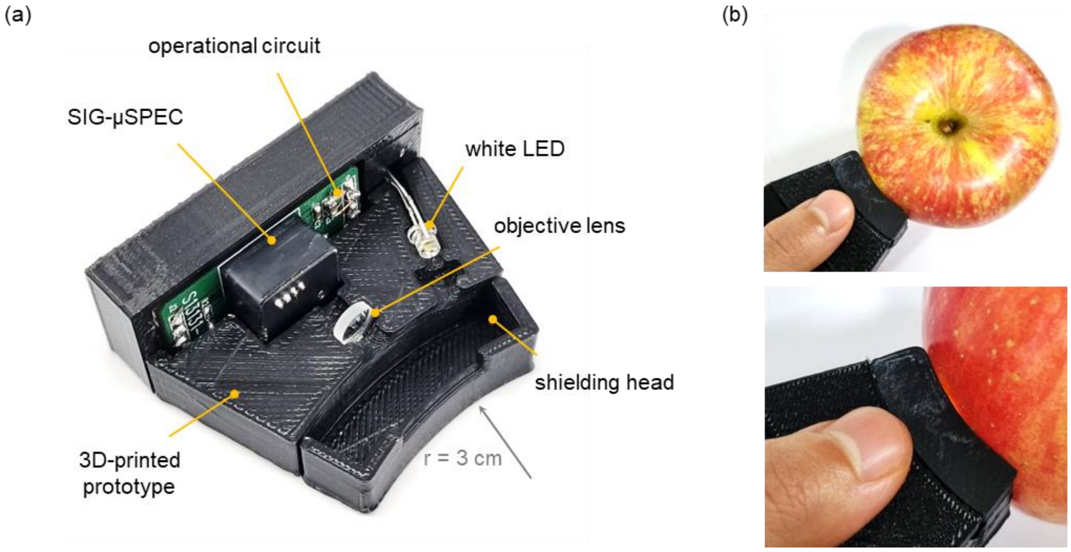

Fig. S5 Handheld module for fruit reflectance spectrum measurement. (a) An optical image of the handheld prototype module. (b) Optical images of the reflectance spectrum measurement.

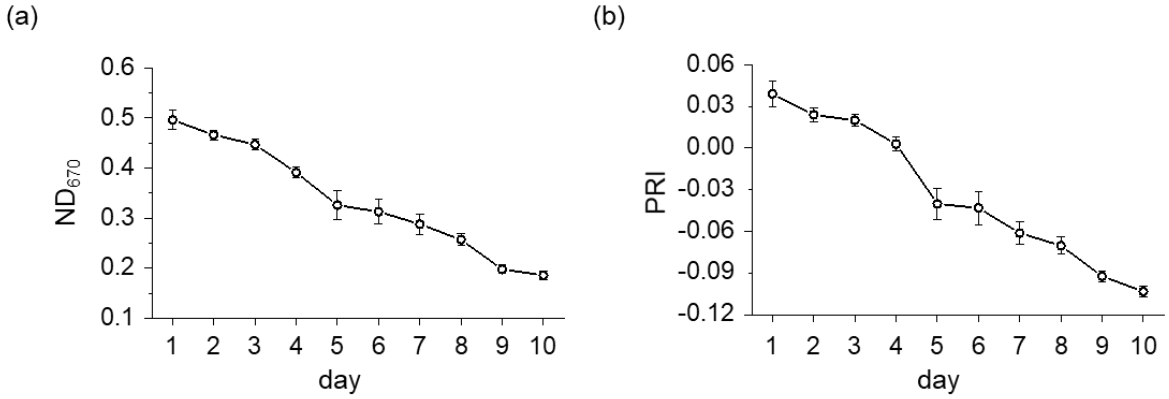

Fig. S6 Quantitative analysis of surface pigment content. (a) Chlorophyll content of apples. Normalized difference vegetation index at 670 nm (ND_{670}) decreased as an apple ripens. (b) Content ratio of carotenoid to chlorophyll. Photochemical reflectance index (PRI) also decreased as apple ripens.

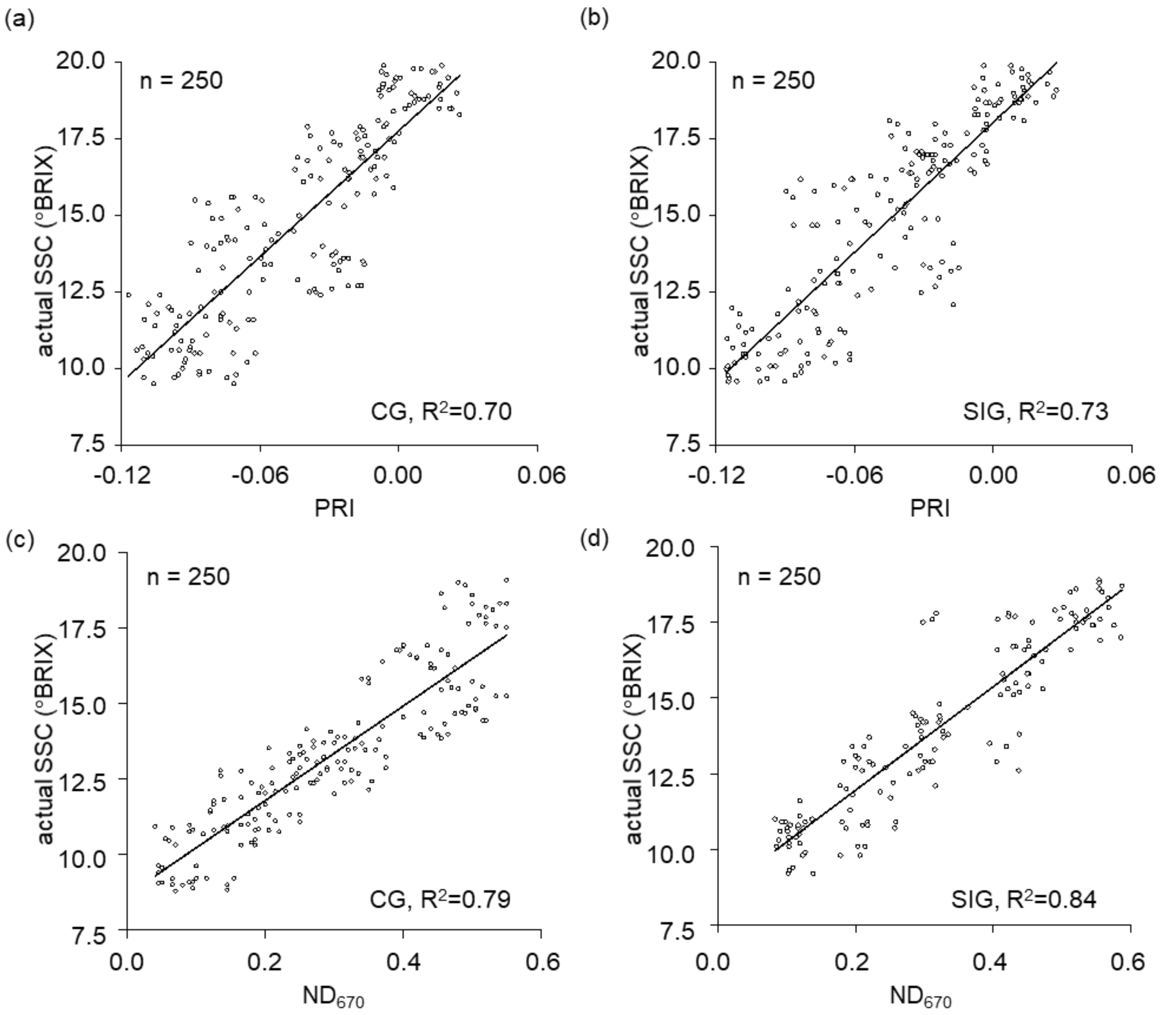

Fig. S7 Linear correlations of the apple reflection spectrum depending on different feature selection methods. (a-b) Linear correlations between PRI and actual SSC level measured by (a) commercial (Hamamatsu Photonics™, C12666MA, concave grating (CG) type) and (b) SIG-μSPEC. The reliability of SIG-μSPEC is slightly higher than CG-μSPEC, with the R^2 of 0.70 and 0.73, respectively. (c-d) Linear correlations between ND_{670} and actual SSC level measured by (c) CG-μSPEC and (d) SIG-μSPEC. The reliability of SIG-μSPEC is further improved up to R^2 of 0.84 through selecting the reflectance ratio of chlorophyll and the actual SSC level (sample size: 250).

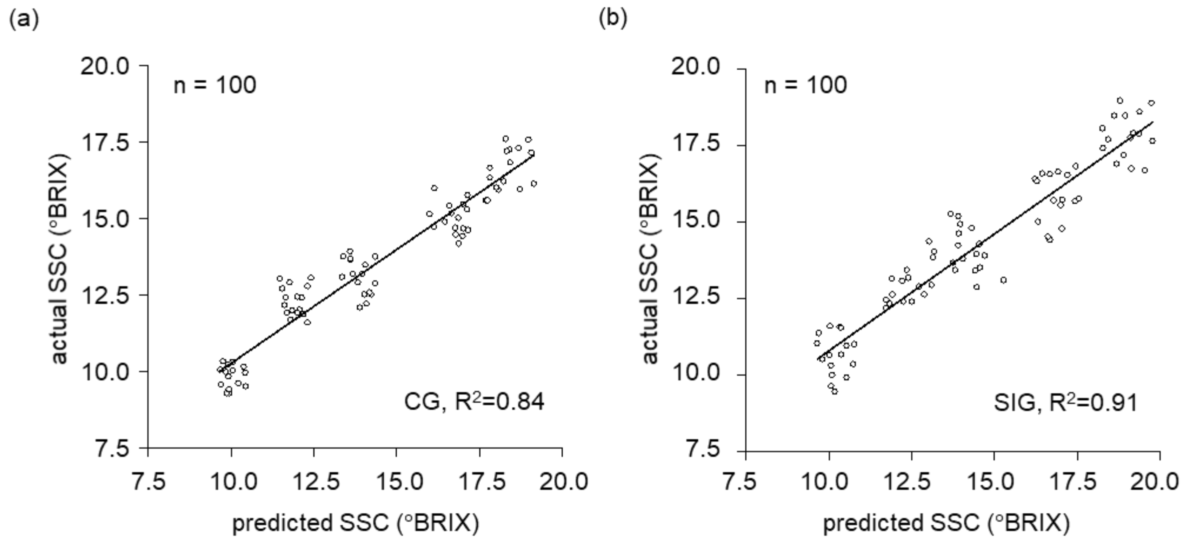

Fig. S8 Linear correlations of the apple reflection spectrum with the feature extraction method. (a-b) Linear correlations between predicted and actual SSC levels using (a) CG-μSPEC and (b) SIG -μSPEC. The reliability of SIG-μSPEC with the feature extraction method achieved an improved R^2 of 0.91, through the establishment of a PLSR model for SSC prediction (sample size: 100).

method abbreviation	signal pre-processing method	R^2	RMSE	RPD
N	no processing	0.76	0.95	1.81
SV	SNV	0.78	0.66	2.00
S1	SNV + 1st derivative	0.79	0.60	1.91
M	MSC	0.82	0.87	2.04
M1	MSC + 1st derivative	0.83	0.90	2.07
O	OSC	0.91	0.61	2.36
O1	OSC + 1st derivative	0.86	0.64	1.98

Table S1 Prediction results depending on pre-processing tools using SIG-μSPEC. The pre-processing tools, including standard normal variate (SNV), multiplicative scattering correction (MSC), orthogonal signal correction (OSC), and the combination of each method with 1st derivative, are evaluated. OSC shows the highest correlation indices among the other pre-processing tools, with the lowest root mean square of error (RMSE), the highest R^2, and the highest RPD. A PLSR model was finally established based on spectrum data with OSC.

correlation index	CG-μSPEC	SIG-μSPEC
R^2	0.84	0.91
RMSEP	0.98	0.61
RPD	1.93	2.36

Table S2 Prediction results of CG-μSPEC and SIG-μSPEC with the feature extraction method. The correlation indices, including RMSE and R^2, indicate higher reliability of SIG-μSPEC compared to CG-μSPEC. Moreover, the RPD of SIG-μSPEC exceeds 2.0, which indicates a high level of prediction accuracy and is comparable to the high-end spectrometers for fruit ripeness testing.
